# Practical Lessons in Nursing

**Published:** 1887-10

**Authors:** 


**Pract'cal Lessons in Nursing. Outlines for the Management of Diet or the Regulation of Food to the Requirements of Health and the Treatment of Disease.** By Edward Tunis Bruen, Assistant Professor of Physical Diagnosis, University of Pennsylvania. Philadelphia: J. B. Lippincott Company. 1887.

This work contains the substance of a course of lectures to the nurses of the training schools of the Philadelphia University of Pennsylvania, and Woman's Hospital. The author has successfully subordinated the scientific aspect of the subject in the presentation of valuable suggestions in the selection of food in health as well as disease. The work presents a condensed summary of facts bearing upon the important subject of dietetics, especially to those engaged in nursing the sick, and we commend it to the profession, to assist them in the management of their patients.

